# Multiparametric Ultrasound and Machine Learning for Predicting Renal Scarring in Children

**DOI:** 10.3390/diagnostics16091311

**Published:** 2026-04-27

**Authors:** Zeynep Ayvat Ocal, Ozgur Ozdemir Simsek, Cemal Bilir, Hasan Turan

**Affiliations:** 1 Department of Radiology, Faculty of Medicine, Çiğli Training and Research Hospital, Bakırçay University, İzmir 35620, Türkiye; 2 Department of Pediatric Nephrology, Faculty of Medicine, Çiğli Training and Research Hospital, Bakırçay University, İzmir 35620, Türkiye; ozgur_ozdemir_07@hotmail.com; 3 Department of Pediatric Surgery, Faculty of Medicine, Çiğli Training and Research Hospital, Bakırçay University, İzmir 35620, Türkiye; cmlbilir@gmail.com; 4 Department of Pediatric Urology, Faculty of Medicine, Çiğli Training and Research Hospital, Bakırçay University, İzmir 35620, Türkiye; hasanturan911@gmail.com

**Keywords:** DMSA, shear wave elastography, renal scarring, ultrasonography

## Abstract

**Background/Objectives**: Renal scarring in children is linked to long-term complications, including hypertension and chronic kidney disease. Although dimercaptosuccinic acid (DMSA) scintigraphy is the reference standard, routine use is limited due to radiation exposure. This study evaluated whether multiparametric ultrasound combined with machine learning could predict DMSA-detected renal scarring in pediatric patients. **Methods**: In this retrospective study, 192 children undergoing renal ultrasound and DMSA scintigraphy were included. Renal morphometric and volumetric parameters, along with shear wave elastography, were analyzed. Supervised machine learning models were trained to predict renal scarring. A validated data augmentation framework addressed class imbalance and limited sample size. Model performance was assessed using standard classification metrics. **Results**: Kidney volume indexed to body surface area and the asymmetry index were strongly associated with renal scarring. Elastography alone had limited discriminatory power in conventional analyses but improved predictive performance when incorporated into machine learning models after data augmentation. Ensemble-based models achieved the highest accuracy and area under the receiver operating characteristic curve. **Conclusions**: Multiparametric ultrasound with machine learning shows potential as a noninvasive tool for predicting renal scarring in children. While not a replacement for DMSA scintigraphy, this approach may aid risk stratification and clinical decision-making, potentially reducing unnecessary radiation exposure.

## 1. Introduction

Renal scarring is a major cause of chronic kidney disease in children, and early detection is essential to prevent long-term complications. Recent advances in ultrasound technology and machine learning (ML) offer the potential to enhance non-invasive prediction of renal scarring by integrating multiple ultrasound parameters and identifying complex patterns that may not be evident through conventional analysis. Multiparametric ultrasound, including morphometric, volumetric, and elastography measurements, may provide complementary information for detecting renal damage without radiation exposure [1,2].

Urinary tract infections (UTIs) in early childhood require close follow-up because inadequately managed infections may progress to chronic conditions and lead to long-term renal damage [3,4,5,6,7]. Chronic kidney disease (CKD) is a progressive disorder characterized by irreversible impairment of renal structure and function, with renal fibrosis representing the central pathological hallmark and the main determinant of disease progression toward end-stage renal disease [8,9]. Although renal biopsy remains the reference standard for assessing renal fibrosis, it is invasive, carries risks such as hematoma, and is unsuitable for repeated evaluations, particularly in pediatric patients [10,11,12,13]. Therefore, reliable non-invasive methods are needed to improve diagnosis, monitoring, and long-term management [10].

Real-time two-dimensional shear wave elastography (2D-SWE) is an advanced ultrasound (US) technique that enables non-invasive assessment of tissue elasticity by measuring shear wave propagation. Since fibrosis is a major determinant of tissue stiffness, elastography may provide a cost-effective and clinically applicable tool for evaluating chronic renal disease [14,15,16]. Elastography measurements can be expressed as Young’s modulus (kPa), shear wave velocity (m/s), or strain ratio [7]. While elastography is widely used in the liver—where tissue stiffness increases with fibrosis—renal evaluation is more challenging due to the kidney’s heterogeneous, compartmentalized, and anisotropic structure, as well as the limited and variable regions available for quantitative assessment [17,18]. Nevertheless, renal elastography has potential for early detection of renal damage and assessment of disease severity [18].

Conventional ultrasonography lacks scar-specific findings and does not reliably predict renal scarring in early stages. Dimercaptosuccinic acid (DMSA) scintigraphy is currently the reference imaging modality for detecting renal scarring, but it involves ionizing radiation and does not provide quantitative information regarding tissue stiffness. Because renal fibrosis underlies renal scarring and influences tissue elasticity, we hypothesized that kidneys with DMSA-confirmed scarring would show increased stiffness on 2D-SWE compared with non-scarred kidneys. Additionally, we hypothesized that elastography-derived stiffness values would be associated with renal scarring. Accordingly, this study aimed to assess renal tissue stiffness using 2D-SWE in kidneys with and without DMSA-detected scarring and to evaluate the role of elastography as a non-invasive marker of renal fibrosis.

## 2. Methods

### 2.1. Study Design and Participants

This retrospective study included pediatric patients who underwent renal ultrasonography and were clinically evaluated for the presence or absence of renal scarring detected on dimercaptosuccinic acid (DMSA) scintigraphy. Written informed consent was obtained from the parents or legal guardians of all participants. The study was conducted in accordance with the Declaration of Helsinki, and all data were anonymized prior to analysis. Institutional Review Board approval was obtained before study initiation (Approval No: 2374, Date: 10 September 2025).

Demographic data, including age and sex, were recorded for all participants. Morphometric variables were derived from standardized sonographic measurements obtained by experienced, European board-certified radiologists using identical imaging protocols in patients with and without DMSA-confirmed renal scars. While the retrospective nature of the study resulted in a maximum interval of six months between DMSA and ultrasound examinations, renal scars were considered chronic structural changes, minimizing the risk of significant temporal bias during this window to minimize confounding effects. Elastography examinations were performed only in patients without active urinary tract infection and with normal renal Doppler findings.

Patients with renal neoplasms, hydronephrosis, congenital renal anomalies, or any signs of acquired renal disease were excluded from the study.

Both kidneys were evaluated for linear dimensions, cortical thickness, and elastographic parameters. Renal volume was calculated assuming an ellipsoidal shape using the formula:

Renal volume = L × W × H × 0.523

where L represents kidney length, W transverse width, and H anteroposterior diameter. To account for individual differences in body size, all volumetric parameters were normalized to body surface area (BSA), calculated according to the Haycock formula:

BSA (m^2^) = 0.024265 × Height (cm)^0.3964^ × Weight (kg)^0.5378^

Absolute kidney volume (KVOL) and BSA-normalized kidney volume (KVOL_BSA) were calculated for each participant.

Inter-renal asymmetry was quantified using an asymmetry index defined as:

Asymmetry index (AI) = (Left kidney volume − Right kidney volume)/Mean kidney volume

Positive values indicated relatively larger left kidneys, whereas negative values indicated larger right kidneys. This continuous ratio allowed detection of subtle unilateral volume reductions independent of overall body size, potentially reflecting renal scarring.

### 2.2. Ultrasonography and Shear Wave Elastography

All participants were informed about the procedure prior to examination. Ultrasonographic and elastographic measurements were performed with the patient in the lateral decubitus position during deep inspiration, with the upper arm positioned above the head to optimize the acoustic window.

**Figure 1:**
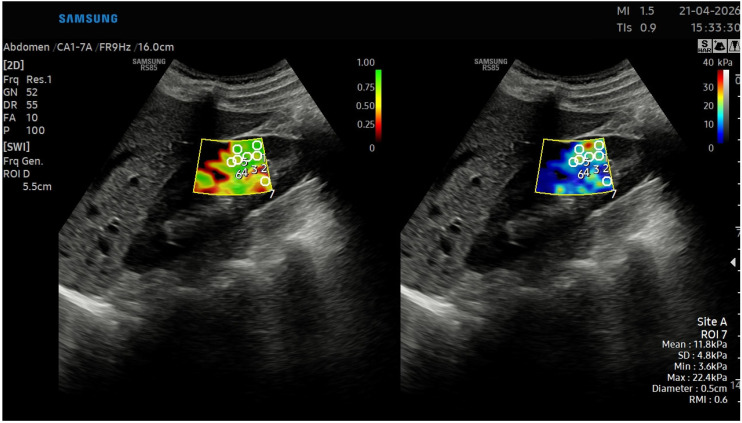
Elastographic measurements and region-of-interest (ROI) placement within the renal cortex.

Examinations were conducted using a two-dimensional shear wave velocity–based elastography system (RS85 Samsung, Seoul, South Korea) equipped with a convex transducer (C5-1, 2–5 MHz). As illustrated in Figure 1, eight to ten region-of-interest (ROI) boxes were placed within the renal cortex of each kidney, evenly distributed across the upper, middle, and lower poles. ROI size was standardized between 0.3 cm and 0.8 cm depending on renal parenchymal thickness.

To minimize anisotropy and cortical thinning effects, ROIs were strictly localized within the renal cortex, ensuring an IQR/M ratio of <30% for all included measurements.

Measurements were obtained with minimal transducer pressure, avoiding proximity to the renal capsule and collecting system. Elastography parameters, including median stiffness values, interquartile range (IQR), and IQR-to-median ratio, were recorded. All measurements were performed by a single experienced radiologist blinded to the patients’ DMSA findings. Mean stiffness values expressed in kilopascals (kPa) were calculated for each kidney, and the procedure was subsequently repeated for the contralateral side.

Renal elastography examinations were scheduled within six months following the DMSA scan. Conventional sonographic measurements included longitudinal and transverse dimensions and parenchymal thickness of each kidney. Patient age, sex, height, and weight were recorded. Data affected by motion or breathing artifacts were excluded from analysis.

### 2.3. Data Augmentation and Machine Learning Preparation

Given the limited dataset size and mild class imbalance favoring the non-scar group, a two-stage data augmentation strategy was implemented to enhance the robustness of subsequent analyses. The dataset was first split into training and test sets using an 80:20 ratio. All data augmentation (Gaussian noise) and SMOTE synthetic oversampling were applied exclusively to the training set. The test set was kept strictly independent and untouched by any augmentation or synthetic data generation.

In the first stage, Gaussian noise was added to continuous variables to simulate physiologically plausible variability while preserving the original data structure. Noise was generated with zero mean and a standard deviation equal to 2% of the empirical standard deviation of each variable. A conservative 2% SD Gaussian noise was selected to introduce minor measurement variability without altering the underlying physiological data distribution. Data consistency was assessed using similarity metrics.

In the second stage, class imbalance was addressed using the Synthetic Minority Oversampling Technique (SMOTE), which generates synthetic samples by interpolating between nearest neighbors in feature space. This process resulted in a balanced 1:1 ratio between scar and non-scar groups. Preservation of the statistical and geometric properties of the data was evaluated using Principal Component Analysis (PCA), t-distributed Stochastic Neighbor Embedding (t-SNE), and Mahalanobis distance analysis, confirming that augmented samples remained within acceptable multivariate limits [19,20,21]. Mahalanobis distance was used as a multivariate measure to assess whether synthetic samples remained within the distribution of the original dataset.

Following data augmentation, the finalized dataset was used to train supervised machine learning classifiers, including logistic regression, random forest, XGBoost, LightGBM, and CatBoost models. To optimize performance and prevent overfitting, hyperparameter tuning was performed, including the use of L2 regularization for the logistic regression model. Feature standardization to zero mean and unit variance was applied prior to model training. Feature selection was guided by clinical relevance, statistical significance, and domain knowledge. Model performance was assessed using accuracy, precision, recall, and F1-score metrics with five-fold cross-validation [21].

### 2.4. Statistical Analysis

All statistical analyses were performed using Python software (version 3.11; Python Software Foundation, Wilmington, DE, USA).Numerical analyses were conducted using the pandas, numpy, and scipy libraries, while receiver operating characteristic (ROC) analyses, SMOTE implementation, and machine learning modeling were performed using scikit-learn. Data visualization was carried out using matplotlib.

Data completeness and physiological plausibility were assessed prior to analysis. Normality of continuous variables was evaluated using the Shapiro–Wilk test. Variables with normal distribution were compared using Welch’s *t*-test, whereas non-normally distributed variables were analyzed using the Mann–Whitney U test. Categorical variables were compared using the chi-square test. ROC curve analysis was used to evaluate the diagnostic performance of significant variables, with the area under the curve (AUC), sensitivity, and specificity calculated at optimal cut-off values determined by Youden’s index. A *p*-value < 0.05 was considered statistically significant.

## 3. Results

### 3.1. Prediction of Renal Scarring on DMSA Using Multiparametric Ultrasound and Shear Wave Elastography

A total of 192 pediatric patients were included in the study, comprising 42 patients in the scar-positive group and 150 patients in the scar-negative group. The median age of the overall cohort was 10 years (interquartile range [IQR]: 8–13 years). There was no significant difference in age or sex distribution between the two groups (*p* > 0.05).

Comparisons of demographic, morphometric, volumetric, and elastographic parameters between the scar-positive and scar-negative groups are summarized in Table 1. Age, body weight, height, body mass index (BMI), and body surface area (BSA) did not differ significantly between groups (*p* > 0.05).

Morphometric and volumetric renal parameters, including kidney width (K2), cortical thickness (CT), kidney volume (KVOL), kidney volume indexed to body surface area (KVOL_BSA), and the asymmetry index (AI), were significantly lower in the scar-positive group compared with the scar-negative group (*p* < 0.001 for all; Table 1).

Elastography-derived parameters, including median stiffness values, interquartile range (IQR), and elastography percentage, did not differ significantly between the two groups (*p* > 0.05; Table 1).

**Figure 2:**
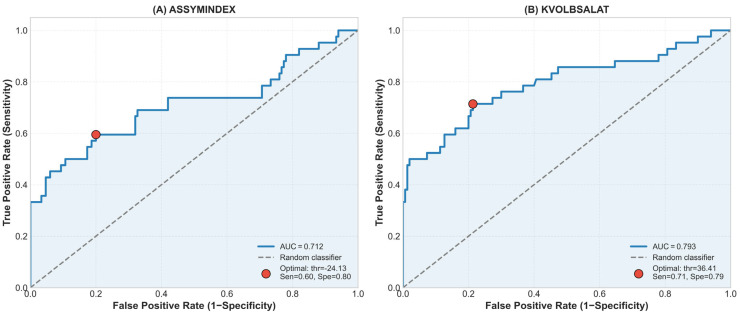
Receiver operating characteristic (ROC) curves for asymmetry index and KVOL_BSA.

Receiver operating characteristic (ROC) analysis was performed for parameters demonstrating significant group differences. KVOL_BSA showed an area under the curve (AUC) of 0.79, while the asymmetry index demonstrated an AUC of 0.71. Sensitivity and specificity values at optimal cut-off points are presented in Figure 2.

### 3.2. Prediction of Renal Scarring on DMSA Using Machine Learning

The initial dataset consisted of 153 training samples and 39 test samples. The training dataset was imbalanced, with a predominance of non-scar cases. Data augmentation procedures were applied exclusively to the training dataset, while the test dataset remained unchanged throughout all analyses.

**Figure 3:**
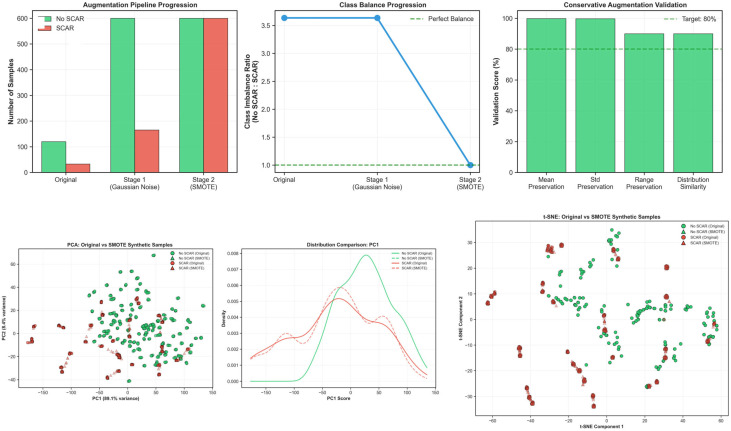
Data augmentation pipeline and validation of synthetic samples using PCA, t-SNE, and Mahalanobis distance analysis.

Following Gaussian-based augmentation, the training dataset was expanded to 765 samples. Distributional similarity between original and augmented data was preserved, as demonstrated by similarity metrics and distribution analyses (Figure 3). Subsequent application of the Synthetic Minority Oversampling Technique (SMOTE) resulted in a balanced training dataset comprising 1200 samples.

Validation of the augmented dataset using Mahalanobis distance analysis revealed no outliers among synthetic samples. Principal component analysis (PCA) and t-distributed stochastic neighbor embedding (t-SNE) demonstrated substantial overlap between original and synthetic samples, confirming preservation of the intrinsic data structure (Figure 3). The mean ± SD Mahalanobis distance values were 3.149 ± 0.882 and 2.994 ± 0.855 for original and synthetic data, respectively.

After feature standardization and selection, supervised machine learning models—including logistic regression, random forest, XGBoost, LightGBM, and CatBoost—were trained using five-fold cross-validation. Similar approaches using transfer learning in ultrasound images have demonstrated improved classification performance even with limited datasets [22]. Model performance metrics, including accuracy, precision, recall, and F1-score, are presented in Table 2.

**Figure 4:**
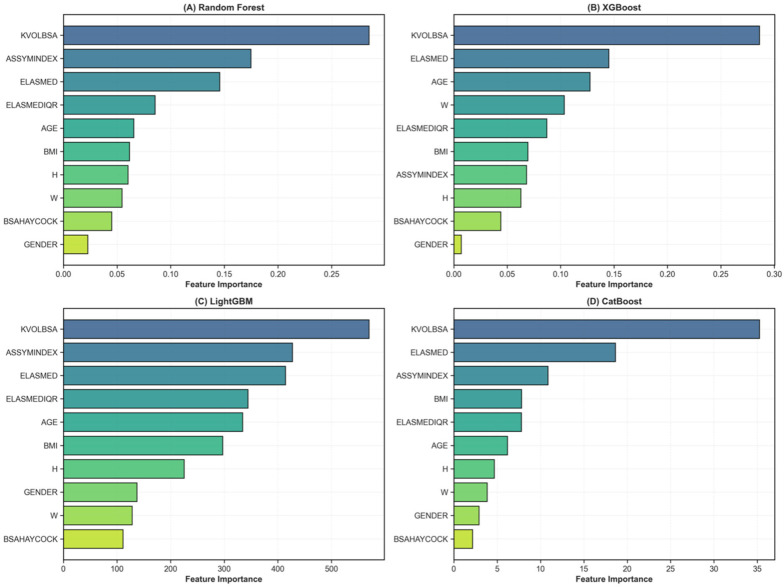
Feature importance rankings across tree-based classification models.

Feature importance rankings derived from tree-based models are summarized in Table 3 and visualized in Figure 4. Elastography-related variables ranked among the top predictors across multiple models following data augmentation.

A discrepancy was observed between the near-perfect cross-validation scores (~0.99) and the performance on the independent test set. While the high cross-validation metrics reflect the model’s internal consistency within the synthetically augmented training environment, the independent test set (comprising 39 unseen cases) provides a more realistic and unbiased estimate of the model’s clinical generalizability.

**Figure 5:**
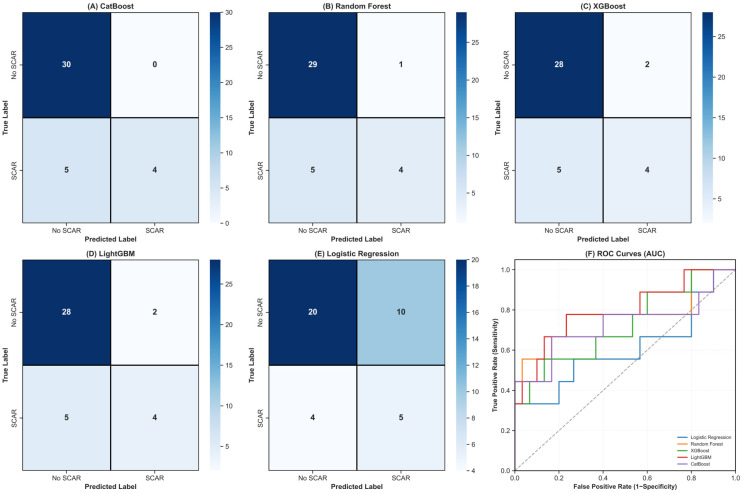
Confusion matrices and comparative ROC curve analysis of the evaluated classification models on the independent test set. Panels (**A**–**E**) show the confusion matrices for CatBoost, Random Forest, XGBoost, LightGBM, and Logistic Regression, respectively. The image displayed in the (**F**) illustrates the ROC curves of all models on the test set.

Overall classification performance of the trained models on the independent test dataset is illustrated in Figure 5.

## 4. Discussion

This study evaluated renal morphometric, volumetric, and elastographic parameters for the detection of renal scarring in a pediatric population and investigated the added value of machine learning–based models supported by rigorously validated data augmentation techniques. The findings indicate that volumetric and structural renal parameters—particularly kidney volume indexed to body surface area (KVOL_BSA) and the asymmetry index—are significantly associated with renal scarring, whereas elastography measurements alone demonstrated limited discriminatory power when assessed using conventional statistical analyses. Notably, elastography-derived features gained predictive relevance when incorporated into machine learning models following dataset expansion.

The observed reductions in kidney volume, cortical thickness, and indexed volumetric parameters in scar-positive patients are consistent with the established pathophysiology of renal scarring, which involves irreversible parenchymal loss and impaired renal growth [23,24]. Indexing kidney volume to body surface area proved particularly informative in this pediatric cohort by minimizing the confounding effects of age-related somatic growth. The moderate-to-good diagnostic performance of KVOL_BSA observed in this study supports its potential role as a non-invasive imaging biomarker for renal scarring.

The significant differences observed in the asymmetry index likely reflect unilateral or disproportionate renal damage, a characteristic feature of reflux nephropathy and post-infectious renal scarring in children [25]. However, the relatively modest sensitivity of this parameter suggests that asymmetry-based metrics alone may not be sufficient for reliable screening and are better suited for inclusion within multivariable prediction models rather than as standalone indicators.

The wide range observed in the Asymmetry Index (AI) reflects cases of severe unilateral renal atrophy and contralateral compensatory hypertrophy. To mitigate the impact of these extreme but clinically relevant values on model training, a robust scaling method was employed. By utilizing Standard Scaler, we ensured that the model remained stable and generalizable, effectively preventing these valid outliers from disproportionately influencing the machine learning results.

In contrast, elastography parameters did not differ significantly between scar-positive and scar-negative groups in univariate analyses. This finding aligns with previous studies reporting inconsistent associations between renal stiffness and fibrosis when assessed using ultrasound elastography. In shear wave imaging, tissue elasticity is estimated by measuring shear wave propagation speed, which may be influenced not only by interstitial fibrosis but also by renal blood flow, tissue perfusion, and hemodynamic factors [26]. As a result, prior investigations have reported positive, negative, or absent correlations between renal elasticity and fibrosis, underscoring the complexity of interpreting elastography findings in native renal tissue.

Despite these methodological refinements, we acknowledge that the reliance on synthetic data augmentation (SMOTE) to address class imbalance remains a limitation. While this technique allowed for more effective model training, it may contribute to a potentially optimistic estimation of performance compared to a purely clinical, non-augmented dataset. Furthermore, the modest sensitivity of our models suggests that at this stage, this machine learning approach serves better as a “ruling-in” confirmatory tool rather than a standalone screening method. Future validation on larger, multi-center cohorts without synthetic expansion is necessary to ensure the clinical generalizability of these findings.

Strain imaging, which estimates tissue elasticity based on tissue deformation induced by external compression or physiological motion, has shown relatively better performance in superficial organs and transplanted kidneys. However, its diagnostic utility in native kidneys is limited by restricted compression transmission, depth-related signal attenuation, and anatomical constraints. These technical and physiological limitations likely contribute to the lack of significant elastography differences observed between scar-positive and scar-negative groups in the present study when elastography was evaluated in isolation.

In our study, the prominent role of elastography parameters in machine learning models—despite a lack of significance in univariate statistical analysis—was further corroborated by SHAP (SHapley Additive exPlanations) analysis. This suggests that machine learning algorithms, particularly tree-based ensembles, are capable of capturing complex, non-linear interactions within the renal tissue stiffness data that traditional linear methods may overlook. While these findings are exploratory given the sample size, they highlight a promising direction for utilizing multiparametric ultrasound features in predictive modeling.

The observed discrepancy between near-perfect cross-validation scores and more modest independent test performance is a recognized effect of synthetic data augmentation. While SMOTE was essential to address class imbalance, it can lead to class boundary inflation. To counter this, we employed robust tree-based ensemble models and prioritized the independent test set as the primary validator of real-world generalizability.

Importantly, after dataset expansion using Gaussian-based augmentation and SMOTE-based class balancing, elastography-derived features emerged as relevant predictors across multiple tree-based machine learning models. While such large-scale augmentation can introduce limitations and lead to optimistic performance [27,28,29], we ensured that our independent test set (*n* = 39) consisted only of original, non-synthetic data to maintain real-world reliability [6,30]. This observation suggests that elastography contains clinically meaningful information that may be subtle, non-linear, and insufficiently captured in small or imbalanced datasets using traditional statistical approaches. The validated data augmentation framework employed in this study enhanced feature representation while preserving the intrinsic statistical structure of the original dataset, as confirmed by Mahalanobis distance analysis, principal component analysis, and t-distributed stochastic neighbor embedding visualizations.

Machine learning models, particularly ensemble and gradient boosting algorithms, demonstrated robust discrimination between scar and no-scar groups following data augmentation. These findings are consistent with previous reports highlighting the effectiveness of such models in capturing complex feature interactions in medical imaging datasets [31]. Nevertheless, several limitations warrant consideration. The retrospective design and strict inclusion criteria limited the original sample size, necessitating data augmentation. The subsequent performance gap between the linear logistic regression and the more robust tree-based ensemble models suggests the presence of complex, non-linear relationships within the multiparametric ultrasound features. Although extensive internal validation was performed, external validation in independent cohorts is required to confirm generalizability. Additionally, the lack of standardized renal elastography protocols and the influence of physiological confounders such as renal perfusion and blood pressure changes may affect reproducibility across centers.

Overall, the present findings suggest that renal scarring in children is predominantly associated with volumetric and structural alterations, while elastography-related changes appear to be more complex and less readily detectable using conventional analytical approaches. The increased contribution of elastography-derived features within machine learning models highlights the potential value of advanced analytical techniques for extracting clinically relevant information from multiparametric ultrasound data, providing a foundation for future prospective and multicenter investigations.

Finally, our study is limited by the single-observer design without formal reproducibility assessment. To mitigate potential bias, the radiologist was blinded to the DMSA results, and a strict quality protocol involving 8–10 ROIs per kidney and an IQR/M < 30% was maintained. A primary methodological limitation is the heavy reliance on synthetic data generation (SMOTE and Gaussian noise) to address class imbalance. Although this expansion was necessary for model training, it may lead to inherently optimistic performance metrics during cross-validation. To ensure clinical validity, we prioritized results from an independent, non-augmented test set (*n* = 39), which revealed a relatively low sensitivity (0.56) compared to high specificity. This reflects a conservative thresholding strategy aimed at minimizing false-positive diagnoses in children. While our models currently serve as a reliable ‘ruling-in’ adjunct, future studies with larger, naturally balanced datasets will focus on optimizing probability thresholds to enhance sensitivity for broader screening purposes. Furthermore, we acknowledge the 6-month interval between DMSA and ultrasound; however, since renal scars represent permanent structural changes, their characteristics are not expected to change significantly within this timeframe. Lastly, as a single-center study using a specific US platform, the external validity across different vendors remains to be established. Therefore, these findings should be viewed as supportive tools rather than standalone alternatives to scintigraphy.

## 5. Conclusions

This study demonstrates that renal morphometric and volumetric parameters, particularly kidney volume indexed to body surface area and the asymmetry index, may be closely associated with renal scarring in children. While elastography alone showed limited diagnostic performance in conventional analyses, its value emerged when integrated into machine learning models using validated data augmentation strategies. These findings highlight the potential of combining multiparametric ultrasound with advanced analytical methods as a non-invasive strategy for evaluating renal scarring in pediatric populations. However, due to the relatively small size of the independent test set and the observed modest sensitivity, these results should be interpreted as exploratory and hypothesis-generating. While our machine learning models show promise as a non-invasive adjunct, they are not intended to replace DMSA scintigraphy at this stage. Further prospective, multi-center trials incorporating larger, non-augmented cohorts are essential before these models can be adopted as robust diagnostic tools in routine clinical practice.

## Figures and Tables

**Table 1:** Demographic and ultrasound parameters of patients with and without renal scarring.

Parameter	All Patients (*n* = 192)	Scar (+) (*n* = 42)	Scar (−) (*n* = 150)	*p* Value
Age (years)	10 (8–13)	10 (7.2–12.7)	10 (8–13)	>0.05
Gender (M:F)	121:71	26:16	95:55	>0.05
Weight (kg)	39 (28.5–51)	39 (27.5–51.7)	39 (29.2–50)	>0.05
Height (cm)	144.6 ± 16.7 (110–180)	145.4 ± 18.5 (112–180)	144.4 ± 16.3 (110–180)	>0.05
BMI (kg/m^2^)	18.5 (15.8–20.8)	18.5 (16.1–20.3)	18.7 (15.6–20.8)	>0.05
BSA (m^2^)	1.2 (1–1.4)	1.2 (1–1.5)	1.2 (1–1.4)	>0.05
K1 (mm)	91.7 ± 15.0 (39–128)	81.1 ± 16.2 (39–115)	94.6 ± 13.3 (66–128)	<0.001
K2 (mm)	37 (33–43)	32.5 (27–40)	38 (34–43.75)	<0.001
CT (mm)	14 (12–16)	12 (10–14)	15 (13–16)	<0.001
KVOL (cm^3^)	52.0 (35.1–68.6)	32.8 (24.2–53.1)	56.1 (42.2–70.0)	<0.001
KVOL/BSA (cm^3^/m^2^)	42.4 (33.8–52.8)	28.1 (17.8–38.2)	46.6 (36.9–54.8)	<0.001
AI	6.2 (−179.6–112.7)	31.9 (−88.2–33.1)	17 (15.7–45.7)	<0.001
KVOL/BSA (AI-adjusted) (cm^3^/m^2^)	42.2 (33.3–52.2)	29.5 (18.7–38.5)	46 (37–53.5)	<0.001
Elastography median (kPa)	14.3 (9.2–17.2)	13.3 (7.9–17.2)	14.6 (10.1–17.1)	>0.05
Elastography IQR (kPa)	3.2 (2.2–4.1)	2.7 (1.7–4)	3.3 (2.2–4.1)	>0.05
Elastography %	0.254 (0.211–0.275)	0.268 (0.248–0.296)	0.254 (0.207–0.276)	>0.05

***Values are presented as median (interquartile range) or mean ± standard deviation. Statistical tests:*** Mann–Whitney U test, Welch’s *t*-test, Chi-square test. Abbreviations: BMI: Body mass index; BSA: Body surface area; K1: Kidney length; K2: Kidney width; CT: Cortical thickness; KVOL: Kidney volume; AI: Asymmetry index; AI-adjusted KVOL/BSA: Kidney volume indexed to BSA and adjusted for asymmetry.

**Table 2:** Cross-validation performance of the machine learning models.

Model	CV Accuracy	CV Precision	CV Recall	CV F1-Score	CV ROC-AUC
LightGBM	0.996 ± 0.004	0.998 ± 0.003	0.993 ± 0.008	0.996 ± 0.004	0.999 ± 0.001
Random Forest	0.995 ± 0.004	0.998 ± 0.003	0.992 ± 0.008	0.995 ± 0.004	1.000 ± 0.000
XGBoost	0.994 ± 0.003	0.997 ± 0.004	0.992 ± 0.008	0.994 ± 0.003	0.999 ± 0.001
CatBoost	0.988 ± 0.005	0.987 ± 0.007	0.990 ± 0.006	0.988 ± 0.005	0.999 ± 0.001
Logistic Regression	0.789 ± 0.029	0.793 ± 0.028	0.783 ± 0.050	0.787 ± 0.033	0.859 ± 0.011

**Table 3:** Performance metrics of the machine learning models for predicting renal scarring.

Model	Accuracy	Sensitivity	Specificity	Precision (PPV)	NPV	F1-Score
CatBoost	0.87	0.56	0.97	0.83	0.88	0.67
Random Forest	0.85	0.44	0.97	0.80	0.85	0.57
XGBoost	0.82	0.44	0.93	0.67	0.85	0.53
LightGBM	0.82	0.44	0.93	0.67	0.85	0.53
Logistic Regression	0.64	0.56	0.67	0.33	0.83	0.42

## Data Availability

The data presented in this study are available on request from the corresponding author due to ethical restrictions regarding pediatric patient data.

## Abbreviations

UTI	Urinary tract infection
DMSA	Dimercaptosuccinic acid
CKD	Chronic kidney disease
2D-SWE	Two-dimensional shear wave elastography
YM	Young’s modulus
US	Ultrasound
BSA	Body surface area
KVOL	Absolute kidney volume
KVOL_BSA	BSA-normalized kidney volume
ROC	Receiver operating characteristic
AUC	Area under the curve
SMOTE	Synthetic minority oversampling technique
PCA	Principal component analysis
t-SNE	t-distributed stochastic neighbor embedding
BMI	Body mass index
CT	Cortical thickness
